# Caregivers’ anxiety and perception of their children’s wellbeing: a year into the COVID-19 pandemic

**DOI:** 10.3389/fpsyg.2023.1115322

**Published:** 2023-05-18

**Authors:** Judith Pena-Shaff, Ashtyn Heckart, Caroline Mannion, Kayla Barry

**Affiliations:** Developmental and Educational Psychology Research Team, Department of Psychology, Ithaca College, Ithaca, NY, United States

**Keywords:** COVID-19 pandemic, youth parents’ anxiety, children’s emotional states, children’s coping, psychological wellbeing

## Abstract

The COVID-19 pandemic and related social restrictions disrupted family routines affecting millions of youths and their caregivers worldwide. This study explored the relationship between caregivers’ anxiety and their children’s emotional states 1 year after COVID-19, as well as differences between caregivers’ perceptions of their children’s emotional states and reality. Sixty-eight caregiver-child pairs completed an online survey between March 31 and May 31, 2021. Our analysis showed positive correlations between caregivers’ anxiety and children’s wellbeing, worries about children’s use of time, and a variety of negative emotional states in their children. Caregivers’ anxiety about their children’s wellbeing was negatively correlated with their children’s perceptions of self-control. Caregivers’ anxiety about their personal wellbeing was negatively correlated with children’s feelings of busyness and positively associated to their children’s fear. Caregivers’ perception of COVID-19 as a challenging experience was positively associated with some of their children’s negative emotions. Overall, caregivers were accurate about children’s emotional experiences in the previous 3 months with some exceptions: their children felt lonelier and more worried about schoolwork and grades than their caregivers realized. These findings will help researchers and practitioners further explore the sources of caregivers’ anxiety and their relationship with children’s emotions and stress management as countries move toward a new normal.

## 1. Introduction

Research has revealed the large impact the COVID-19 pandemic and its related life changes has had on family routines, affecting millions of youths and their caregivers worldwide including striking effects on mental health due to prolonged fear and health measures, like social distancing (Jin et al., 2022). This study focused on caregivers’ (those taking parental roles), and youths’ (ages 9–18 years) perceptions of their psychological wellbeing 20 months after COVID-19 social restrictions were first enforced in the United States.

The first known infections of the severe acute respiratory syndrome SARS-CoV-2 causing the coronavirus disease (COVID 19) were identified in Wuhan, Hubei, China, in 2019. In January 2020, the extent of the epidemic in China became apparent to the world as images of this region’s lockdown appeared (Katella, 2021). Soon after, cases were identified in the Philippines, Europe, and the United States. By March 2020, most of the world shut down, with countries closing their borders, schools, and workplaces. People were required to wear masks, social distance, and work/learn from home (Katella, 2021). Adults with children reported an increase in stress and anxiety in all aspects of daily life (American Psychological Association, 2020). In addition to significant levels of stress related to the disease, caregivers also worried about their children’s schooling, the long-term impacts on their social–emotional development and mental health, and about keeping their children occupied during the pandemic (American Psychological Association, 2020). Based on a 2020 APA report, caregivers’ anxiety was related to their children’s overall wellbeing and development, and their activities during lockdowns.

### 1.1. Caregivers’ anxiety and their children’s psychological wellbeing

Disruptions in school and workplace, in addition to COVID-19 fears, have been associated with stressors to children, their caregivers, and the overall family (American Psychological Association, 2020). In addition, confinement of caregivers for a long time without opportunities to pursue personal interests and goals increased conflict and tensions at home for many (Mohler-Kuo et al., 2021). Several studies report on the increased levels of anxiety, depression, and perceived stress in caregivers during the pandemic (American Psychological Association, 2020; Spinelli et al., 2020; Wu et al., 2020; Mohler-Kuo et al., 2021). Wu et al. (2020) found that parents, particularly those with middle or high school children, reported high levels of depression, anxiety, and perceived stress, especially if there were family conflicts. Spinelli et al. (2020) reported that household chaos during the pandemic affected parents’ stress levels. Mohler-Kuo et al. (2021) found that caregivers, particularly mothers, reported higher levels of stress due to the need to “take on greater family or work responsibilities” (p. 6). This study also reported a higher-than-expected level of children’s internet use which also could cause friction between children and their caregivers.

We found only two studies addressing the relationship between caregivers’ anxiety and stress related to COVID-19 and their children’s psychological wellbeing. Schabus and Eigl (2021) in a sample of Austrian youth found that children of higher-anxiety parents worried more about themselves or loved ones getting sick than did those of lower-anxiety parents. In addition, Kerr et al. (2021) found that among caregivers with at least one child aged 12 years or younger, there was a positive correlation between caregivers’ psychological impacts/parental burnout and their perceptions of their children’s stress behaviors. In addition, there was a negative correlation between these and their children’ positive behaviors. It is important to further explore the relationship between caregivers’ anxiety and their children’s emotional states and coping skills to deal with difficult situations such as those brought about by the COVID-19 pandemic.

### 1.2. Youth psychological wellbeing during COVID-19

Immediately after the pandemic began, several studies investigated how the quarantine and social disruptions psychologically affected youth (Orgilés et al., 2000; Buzzi et al., 2020; Liu et al., 2020; Schabus and Eigl, 2021; Tang et al., 2021; Jin et al., 2022). These studies report increased rates of loneliness (Orgilés et al., 2000; Schabus and Eigl, 2021), boredom (Orgilés et al., 2000), fear (Orgilés et al., 2000; Buzzi et al., 2020; Jin et al., 2022), irritability and anger (Orgilés et al., 2000; Schabus and Eigl, 2021), anxiety sensitivity (Jin et al., 2022), and depression (Schabus and Eigl, 2021; Tang et al., 2021). For example, Buzzi et al. (2020) found, in an impromptu study among youth in Italy, that the COVID-19 outbreak had impacted not only youth emotions and lifestyle but also their relationships with parents and peers. Likewise, Orgilés et al. (2000) study on the psychological impact of the COVID-19 social restrictions in children and adolescents in Italy and Spain, found that parents perceived changes in their children’s emotional states such as increased boredom, difficulty concentrating, nervousness, loneliness, and worries since social restrictions were imposed. These problems seemed to be exacerbated by difficult family dynamics and higher caregiver stress levels. Schabus and Eigl (2021) study with Austrian children and adolescents presented “an alarming picture of psychosocial health among children and adolescents, highlighting the acute need for action” (p. 9) due to increased feelings of fury, anger, loneliness, and sadness. Due to the abrupt life changes and fears of the pandemic, psychological effects on the wellbeing of caregivers and children are unsurprising. However, humans, and particularly children and adolescents, are highly resilient and able to adapt to changes in their routines and life situations.

### 1.3. Youth coping and resilience during COVID-19’s social restrictions

Some research evidence from early in the pandemic suggests that children’s and adolescents’ psychological wellbeing improved as they became used to the changes in social life brought about by COVID-19. Liu et al. (2020) reported that residents of Wuhan, China, experienced an increase in post-traumatic stress symptoms (PTSS) due to the psychological trauma of COVID-19. However, they also stated that adolescents were less likely to develop mental health disorders than adults and were more likely to bounce back after experiencing negative events related to the pandemic. Similarly, Yang et al. (2020) found that although COVID-19-related trauma had a significant impact on the mental health of high school students in Wuhan, those with higher levels of resilience and positive emotion regulation had overall better mental health than those who did not. Likewise, Commodari and La Rosa (2020) in a survey with Italian youths, who had very strict quarantine rules between March and May 2020, found that even though many reported negative feelings during those months, they also reported new routines such as exercise and engaging in new hobbies that helped them cope. Youth who followed strict personal protective measures and avoided going out in public to reduce their chances of contracting COVID-19, used social media, and talked about COVID-19 with family and friends, felt better equipped to deal with the social changes imposed by the pandemic (Baloran, 2020).

As Yang et al. (2020) stated, positive emotion regulation and resilience interrupted or reduced the psychological trauma youth experienced during the COVID-19 pandemic, helping protect their mental health. Therefore, understanding how youth dealt with difficult situations can help practitioners develop training and interventions to promote resilience and positive coping strategies to ameliorate or prevent negative mental health outcomes from traumatic life disruptions.

When we began to design our study, most of the published studies came from China or European countries (e.g., Spain, Italy, and Austria). To our knowledge, there were very few published studies, if any, with youth from the United States. In addition, most studies reporting on youth’s psychological wellbeing look either at youth’s self-reports or reports from their parents’ perceptions. Based on the experience of one of this study’s authors, we became intrigued by how attuned caregivers were to their children’s emotional states as well as the relationship between caregivers’ anxiety and their children’s emotional experiences. Previous studies used data collected during the first 6 months after social restrictions began. However, as children and adolescents are highly resilient and adapt more easily than adults to new situations, their psychological state could be different after 20 months. Caregivers and their youth might have begun adapting to their new work, school, and family routines, possibly reducing the mental health impact of the COVID-19 pandemic. In addition, by March 2021, vaccines were becoming available in the United States, and some schools began to offer in-person classes, either full time or in hybrid form, with many of them still following safety measures such as mask-wearing and social distancing. Study Purpose and Research Questions.

The purpose of this study was to explore the relationship between caregivers’ anxiety and their children’s emotional states and coping strategies nearly 1 year after the COVID-19 pandemic social disruptions began in the United States. We also explored differences between caregivers’ perceptions of their children’s emotions and how their children felt. The following questions guided our study:

1. Was there a relationship between caregivers’ perceived anxiety and their children’s emotional states?
2. Was there a relationship between caregivers’ perceived anxiety and how their children dealt with difficult or challenging situations?
3. Were there differences between caregivers’ perceptions of their children emotional states and their children’s emotional states?

## 2. Methodology

### 2.1. Participants

Caregivers participating in the study were 18 years old or older with at least one child between the ages of 9 and 18 years. A total of 156 caregivers accessed the survey; 122 completed it. Participants who did not complete the survey were excluded from the study. Some participants provided information for more than one child between the required ages. In those cases, and if child-caregiver were matched, they were counted as two participants. The caregivers’ survey provided a link to the youths’ questionnaire. Caregivers were invited to share the link with their children if they consented to participate in the study. They were also directed to include a personal code to share with their children to allow researchers to pair caregivers with their respective children. One-hundred and twenty-five youth accessed their survey. Before beginning the survey, they were also asked for their consent to participate. Of these, 99 completed most of the survey questions. Researchers were able to pair 68 of these youth with their respective caregivers. We used these caregiver-youth pairs for the analyses. Table 1 presents the sample demographics.

**Table 1:** Caregivers’ and (Child) demographics (*N* = 68).

Variable	%	Variable	%
*Relationship to child*	*Racial identification*
Mother	65	White	74 (71)
Father	31	Black	19 (18)
Stepparent	2	Native American/Alaskan Native	4 (4)
Other (grandparent; foster parent)	2	Asian/Asian American	1 (2)
*Sex*	Other	2 (2)	*Ethnicity*
Female	62.8 (38)	Non-Hispanic	75 (75)
Male	35.5 (57)	Hispanic	18 (22)
Prefer not to say	(5)	Missing	8 (3)
*Age range*	*Education level*
26–30	3	Highschool or GDE	3
31–35	28	Some college, no degree	14
36–40	24	Trade or technical training	4
41–45 (*Mdn*)	15	Associate degree	6
46–50	15	Bachelor’s degree	21
51–55	13	Master’s or higher degree	44
56–60	3	Missing	8
*Marital status*	*Partner’s education*
Single or never married		Highschool or GDE	4
Married or domestic	1	Some college, no degree	13
Partnership		Trade or technical training	5
Divorced	97	Associate degree	15
	Bachelor’s degree	23	Master’s or higher degree	27
	2	It is just me	4
	Missing	8
*Employment status*	*Approximate household income*
Employed	72.5	<$39,999	9
Self-employed	10	$40,000 to $49,999	6
Homemaker	8	$50,000 to $59,999	7
Student	2	$60,000 to $69,999	3
Unemployed	<1	$70,000 to $79,999	7
Missing	6.5	$80,000 to $89,999	9
	$90,000 to $99,999	8	$100,000 to $149,999	16	$150,000 or more	26	Missing	8
*Area of residence*	Type of home
Rural	19	Apartment	22
Urban	26	Duplex	7
Suburban	54	House	69
	Other	1
*Number of children aged 9–18*	*Children mode of schooling*
One	71	Homeschooling	6
Two	22	Hybrid	39
Three	6	100% in-person	12
Four or more	1	100% remote	42
*Caregivers working from home*	
Yes	57
No	12
Hybrid	31

### 2.2. Measures

We developed two surveys (one for caregivers and one for their children) based on the available literature at the time related to the psychological impact of COVID-19 quarantine and social restrictions in China and several European countries were published shortly after the pandemic began (Orgilés et al., 2000; Commodari and La Rosa, 2020). These studies explored the psychological effects within the first 6 months of social restriction. We used Qualtrics to design the online questionnaires. The online surveys conformed to the recommended standards for conducting internet surveys by following the Checklist for Reporting Results of Internet E-Surveys (CHERRIES): The study was approved by our IRB, participants included gave consent after reading the survey cover letter, etc. Participants were recruited through local advertisement, Facebook accounts, and the use of the snowball sampling technique. Surveys were anonymous. Once caregivers completed the survey, they had the opportunity to add their email to be included in a raffle for a $ 100 Visa card. If they accepted to participate in the raffle, the link took them to another URL to maintain anonymity. The surveys were available between the period of March 31 and May 31, 2021.The caregivers’ survey included a total of 71 items, most of them in Likert-scale form, with four open-ended questions. Caregivers’ survey used a simplified version of the survey used by Orgilés et al. (2000) examining the psychological impact of the COVID-19 quarantine in youth from Spain and Italy. The survey included five sections: (1) caregivers’ sociodemographic information and their children’s ages 9–18 years; (2) COVID-19 perceived levels of risk; (3) caregivers’ perceived levels of anxiety; (4) caregiver’s perceptions of how the COVID-19 restrictions in the previous 2 months were affecting their children’s emotional wellbeing and how they thought their children were dealing with difficult situations; and (5) caregivers’ coping methods and social support systems during the previous 2 months.

The youth survey consisted of 64 items, most of them in Likert-scale or dichotomous responses addressing their experiences during the 2 months before the survey. The survey also included two open-ended questions. The survey included the following sections: (1) their emotions and behaviors during the previous 2 months; (2) relationships and social support experienced; (3) their way of dealing with difficult situations; and (4) demographics. The sections about feelings and dealing with difficult situations presented Likert-scale items matching those on the adult survey. Youth selected the answers that best reflected their emotional states; caregivers selected the answers that best reflected their perceptions of their children’s emotional states.

### 2.3. Data analysis

The 12 items from the COVID-19 caregivers anxiety scale were subjected to principal component analysis (PCA) using SPSS Version 21. Inspection of the correlation matrix revealed the presence of many coefficients of 0.3 or above. The Kaiser-Meyer-Oklin value was 0.744, exceeding the recommended value of 0.6 (Kaiser, 1974) and Bartlett’s test of Sphericity reached statistical significance (<0.001), supporting the factorability of the correlation matrix. Principal components analysis revealed three components with eigenvalues at or above 1, explaining 33.6, 16, and 11.7% of the variance, respectively. These three components were retained. Oblimin rotation was performed to aid in the interpretation of these components. The rotation showed several strong loadings with all variables but one loading on only one component. The interpretation of these three components makes sense [Factor 1. caregivers’ anxiety related to their child(ren) overall development and wellbeing; Factor 2. caregivers’ anxiety related to their personal finances, mental and physical health, and health of other family members, not their children; and Factor 3. caregivers’ anxiety related to their children’s use of time]. There was a weak or non-existent positive correlation between the three factors (Factor 1 and 2, *r* = 0.21; Factor 1 and 3, *r* = 0.024; Factor 2 and 3, *r* = 0.067).

Caregivers’ anxiety related to (1) their children’s development and wellbeing (*Child Wellbeing Anxiety*), (2) their personal finances, mental and physical health (*Personal Anxiety*), and (3) anxiety about how children were using their time at home (*Child Time-Use Anxiety*). We conducted correlations between these anxiety factors and children’s emotional states and how children dealt with difficult situations in the previous 2 months. Youth questions about their emotional states were measured using a 5-point Likert scale ranging from 1 (never) to 5 (always). For example, youth were asked “In the last 2 months, how often have you felt scared.” Ten items related to youth emotional states.

For 68 of these parents-child dyads, we calculated 86 correlations between different variables related to caregiver anxiety and youth emotional states. According to Faul et al.’s (2007) software for power analyses, a sensitivity power analysis showed that an *N* of 68 provides 80% power to detect a correlation of *r* = ± 0.33, *p* < 0.05, two-tailed. With a Bonferroni adjustment to the *p*-value for 76 correlations between different variables (*p* = 0.00066), *N* of 68 provides 80% power to detect a difference between two between-subjects conditions of *r* = ± 0.48, *p* < 0.00066, two-tailed.

For 66 of these parents and 66 children, we also calculated 36 correlations between different variables. According to Faul et al.’s (2007) software for power analyses, a sensitivity power analysis showed that an *N* of 66 provides 80% power to detect a correlation of *r* = ± 0.34, *p* < 0.05, two-tailed. With a Bonferroni adjustment to the *p*-value for 36 correlations between different variables (*p* = 0.00139), *N* of 66 provides 80% power to detect a difference between two between-subjects conditions of *d* = ± 0.467, *p* < 0.00139, two-tailed.

Paired-sample t-tests were conducted to explore mean differences between how caregivers perceived their children’s emotional states and how their children experienced them. As in the youth survey, the caregivers’ survey included 11 items using a Likert scale asking them “In the last 2 months, how often has this child felt scared, happy, etc.” According to Faul et al.’s (2007) software for power analyses, a sensitivity power analysis showed that 68 parent–child dyads achieve 80% power to detect a difference between two between-subjects conditions of *d* = ± 0.345, *p* < 0.05, two-tailed. With a Bonferroni adjustment to the *p*-value for 11 paired-samples *t*-tests (*p* = 0.0045), 68 parent–child dyads achieve 80% power to detect a difference between two between-subjects conditions of *d* = ± 0.460, *p* < 0.0045, two-tailed.

## 3. Results

### 3.1. Demographics

Table 1 provides details on the sample demographics. Participants came from 19 states in the United States, predominantly from the Northeast (New York and Massachusetts) and California. The sample was mostly suburban or urban and most lived in houses, not apartments. Almost all caregivers said their children had opportunities to play or be outside during the pandemic.

Caregivers (female = 62.8%) were between the ages of 26 and 60 years (*Median* for age was 3, which corresponds to ages 41–45 years), mostly White (24% identified as Black/African American, Native American, or Asian), non-Hispanic (19% identified as Hispanic). Most participants were married or in a domestic partnership. About 75% of the participants or their partners had at least some college credit and a yearly household income of $80,000 or more. About 70% of participants and their partners were employed for wages or salary, and only about 10–12% were self-employed. The youth sample (Female = 34%; Male = 63%, nonbinary = 3%) were between 9 and 17 years old (*M* = 13.6; *SD* = 2.4).

### 3.2. Caregivers’ anxiety and their children’s emotional states

Table 2 presents the correlational analysis results and descriptive statistics between caregivers’ anxiety (Child Wellbeing Anxiety, Personal Anxiety and Child Time-Use Anxiety) and youth emotional states. Our analysis showed that caregivers’ anxiety related to their children’s development and wellbeing was positively and moderately correlated with youth feeling lonely (*r* = 0.49, *p* < 0.01), scared (*r* = 0.46, *p* < 0.01), hopeless or sad (*r* = 0.45, *p* < 0.01), lonely (*r* = 0.44, *p* < 0.01), angry and bored (*r* = 0.42, *p* < 0.01), and nervous (*r* = 40, *p* < 0.01). There were also positive but weaker correlations between caregivers’ Child Wellbeing Anxiety and their children being worried about their grades (*r* = 0.39, *p* < 0.01) and feeling rushed (*r* = 0.32, *p* < 0.01). Caregivers’ Child Time-Use Anxiety during the past 2 months was positively correlated with most youth negative emotions and weakly but negatively correlated with their children’s sense of happiness (*r* = −264, *p* < 0.05). Caregivers’ Personal Anxiety was negatively correlated with their children being worried about school (*r* = −0.36, *p* < 0.01) but positively correlated with their children feeling scared (*r* = 0.25, *p* < 0.05). With a Bonferroni adjustment to the value of p (*r* = ± 0.483, *p* < 0.00066, two-tailed), only correlations between caregivers’ anxiety related to their children’s development and wellbeing and children’s loneliness remained significant.

**Table 2:** Correlation between caregivers’ anxiety and youth emotional states.

Variable	Mean	*SD*	1	2	3	4	5	6	7	8	9	10	11	12
1. Child Wellbeing Anxiety	16.7	2.11	1											
2. Personal Anxiety	19.3	3.01	0.10	1										
3. Child Time Use Anxiety	9.5	2.3	0.265*	0.040	1									
*Youth reported emotional states*														
4. Rushed	2.7	1.1	0.32^**^	−0.20	0.41^**^	1								
5. Stressed about school	2.7	1.3	0.21	−0.36^**^	0.30^*^	0.70^**^	1							
6 Worried about grades	2.9	1.4	0.39^**^	0.01	0.47^**^	0.68^**^	0.56^**^	1						
7. Bored	2.9	1.3	0.42^**^	−0.01	0.45^**^	0.69^**^	0.53^**^	0.64^**^	1					
8. Lonely	2.7	1.4	0.49^**^	−0.06	0.41^**^	0.73^**^	0.51^**^	0.61^**^	0.74^**^	1				
9. Scared	2.5	1.4	0.46^**^	0.25^*^	0.41^**^	0.66^**^	0.39^**^	0.65^**^	0.71^**^	0.74^**^	1			
10. Nervous	2.7	1.3	0.40^**^	0.06	0.37^**^	0.64^**^	0.55^**^	0.72^**^	0.67^**^	0.68^**^	0.80^**^	1		
11. Angry	2.5	1.1	0.42^**^	−0.01	0.38^**^	0.73^**^	0.55^**^	0.66^**^	0.68^**^	0.73^**^	0.77^**^	0.75^**^	1	
12. Hopeless/sad	2.6	1.3	0.45^**^	−0.10	0.39^**^	0.74^**^	0.63^**^	0.69^**^	0.64^**^	0.79^**^	0.76^**^	0.82^**^	0.77^**^	1
13. Happy	2.7	1.2	0.05	−0.12	−0.26^*^	−0.39^**^	−0.35^**^	−0.21	−0.46^**^	−0.33^**^	−0.27^*^	−0.36^**^	−0.48^**^	−0.31^*^

^**^p < 0.01 level (2-tailed); ^*^p < 0.05 level (2-tailed); *N* = 68.

### 3.3. Caregivers’ anxieties and how their youth dealt with difficult situations

Table 3 presents the correlational analysis results and descriptive statistics between the factors that drove caregivers’ anxiety (Child Wellbeing Anxiety, Personal Anxiety, and Child Time Use Anxiety) and youth perceptions on how they dealt with difficult situations. Our analysis showed that caregivers’ Child Wellbeing Anxiety negatively correlated with youth perception of self-control (*r* = −0.35, *p* < 0.01); in addition, caregivers’ Personal Anxiety was positively associated with their youth asking friends, teachers, and family for help if needed (*r* = 0.25, *p* < 0.05). These results, however, were underpower, as only anything above 0.467 remained significant with a Bonferroni adjustment.

**Table 3:** Correlation between caregivers’ anxiety and youth perceptions of how they dealt with difficult situations.

Variable	Mean	*SD*	1	2	3	4	5	6	7	8
1. Child Wellbeing Anxiety	16.7	2.1	1							
2. Personal Anxiety	19.4	3.0	0.11	1						
3. Child Time Use Anxiety	9.5	2.3	0.29*	0.53	1					
Find different ways to make themselves feel better when things do not go their way	3.0	0.4	−0.15	−0.02	−0.04	1				
Can grow as a person by dealing with difficult situations	3.0	0.5	−0.02	−0.21	0.07	0.41**	1			
Look for new ways to deal with difficult situations	2.9	0.4	−0.13	−0.06	−0.05	0.25*	0.49**	1		
Can control emotional reactions	2.6	0.7	−0.34**	0.17	−0.12	0.15	0.30*	0.24	1	
Only sets goals that they know can achieve without help	2.7	0.6	−0.05	0.03	0.10	0.25*	0.20	0.30*	0.36**	1
Asks friends, teachers, or family when needing help	2.9	0.6	−0.11	0.25*	−0.07	0.23	0.33**	0.40**	0.33**	0.06

**p < 0.01 (2-tailed); *p < 0.05 level (2-tailed), *N* = 66.

### 3.4. Differences between caregivers’ and their youth’s perceptions of youth emotions

Table 4 shows the results of the paired sample t-tests conducted to examine differences in caregivers’ perceptions of their children’s emotional states and their children’s actual emotional states. We found statistically significant differences in perceptions for how worried or stressed children felt about schoolwork (*t* = −3.64, *p* < 0.001, *Cohen’s d* = 0.90) and how bored (*t* = −3.44, *p* < 0.001, *Cohen’s d* = 1.06) children felt in the previous 2 months. This suggests that children felt more worried about schoolwork and were more bored than caregivers realized. In addition, caregivers perceived their children to be less rushed (*t* = −2.43, *p* < 0.05, *Cohen’s d* = 0.75), less worried about grades (*t* = −2.20, *p* < 0.05, *Cohen’s d* = 0.88), and less lonely (*t* = −2.06, *p* < 0.05, *Cohen’s d* = 1.06) than they were. Overall, youth in our sample were more worried about schoolwork and their grades and felt more rushed, lonely, and bored than their caregivers perceived them to be. The percentage of youth who often or always experienced negative emotions was higher than what their caregivers perceived (Table 4).

**Table 4:** Differences between caregivers’ and youth perceptions of youth emotional states.

Variables	Caregivers’ perceptions of child emotional state (*N* = 68)			Youth emotional states (*N* = 68)			Paired-t
	*M*	*SD*	Often/Always %	*M*	*SD*	Often/Always %	
Rushed	2.49	1.02	15	2.71	1.15	22	−2.43*
Stressed about schoolwork	2.26	1.06	22	2.66	1.32	38	−3.64**
Worried about grades	2.69	1.25	31	2.92	1.41	37	−2.20*
Bored	2.5	1.11	28	2.94	1.35	33	−3.44**
Lonely	2.46	1.15	19	2.72	1.36	26	−0.206*
Fearful	2.46	1.16	16	2.49	1.38	16	−0.270
Anxious	2.68	1.13	27	2.74	1.35	29	−0.541
Angry	2.51	1.13	18	2.50	1.127	16	0.148
Sad	2.43	1.08	17	2.57	1.30	24	−1.344
Happy	2.78	1.03	36	2.66	1.17	28	1.09

**p < 0.01 (2-tailed); *p < 0.05 level (2-tailed).

## 4. Discussion

We explored the relationship between caregivers’ anxiety and their children’s emotional states and how youth dealt with difficult situations 20 months after the COVID-19 pandemic social disruptions began in the United States. In addition, we explored differences between caregivers’ perceptions of their children’s emotions and how their children felt.

First, we found that caregivers’ anxiety related to their children’s development and wellbeing was positively and moderately correlated with some negative emotions experienced by their children such as feeling scared, sad, lonely, angry, bored, and nervous, and to a lesser but still significant degree to their children being worried about their grades and feeling rushed. Caregivers’ anxiety over how children spent their time (e.g., screen time) over the previous 2 months was also correlated with most negative emotions in their children and weakly but negatively correlated with their children’s sense of happiness. Caregivers’ anxiety about their own personal matters negatively correlated with children being worried about school but positively correlated with their children feeling scared. It is possible that caregivers’ anxiety about their children’s wellbeing increased if they perceived them experiencing negative emotions such as fear and sadness. Likewise, it is possible that seeing their children spending too much time playing video games or on social media and not enough on school-related work made them anxious about how they were spending their time. These issues could also cause conflicts, exacerbating anxiety in the caregivers and negative emotions in their children. As such, youth negative emotions could also be the result of caregivers’ increased anxiety about their children’s emotions and their use of time and the conflict that could arise from such interactions. It was interesting to see that caregivers’ anxiety related to their personal matters was inversely correlated with their children feeling worried about school. Since causation cannot be assumed, it is impossible to draw conclusions on whether caregivers could focus more on their personal matters because their kids were focused on their studies or whether their children could be more relaxed if their caregivers were not focusing on their schoolwork. Our findings support earlier evidence on the relationship between caregivers’ anxiety and their children’s psychological wellbeing during the early stages of the pandemic. Several studies (e.g., Spinelli et al., 2020; Kerr et al., 2021; Schabus and Eigl, 2021; Suzuki and Hiratani, 2021; Joo and Lee, 2022) have reported the indirect association of COVID-19 parental stress and their children’s internalizing and externalizing behavioral problems. Frustration related to quarantine or social distancing, living with others in confined spaces with restricted social networks, drastic disruptions in daily routines, and increased parenting demands, particularly on mothers, have been associated with parents’ mental health and behaviors predicting children’s outcomes (Joo and Lee, 2022). However, our study revealed that not all types of caregiver anxiety positively associated with their children’s negative emotional states. Still, we can see the dynamic relationship between youth’s psychological wellbeing and caregivers’ anxiety as it relates to their children’s development, wellbeing, and use of time.

We also found that caregivers’ anxiety related to their children’s development and wellbeing negatively correlated with youth perceptions of self-control. As caregivers’ anxiety over their children’s wellbeing increased, their children’s sense of self-control decreased or vice versa. Again, causality cannot be established. We know from earlier studies that the abrupt changes in daily routines brought about by the pandemic increased caregivers’ anxiety levels as children’s schooling moved online and many caregivers had to either work from home or had to leave their children alone while they risked their lives as essential workers (American Psychological Association, 2020). Many caregivers worried about their children’s schooling and academic prospects (American Psychological Association, 2020). Living in a confined space over a long period of time could have increased tensions and undermined youth’s feelings of self-control over their lives and futures. However, caregivers’ personal-related anxiety was positively associated with their youth asking friends, teachers, and other family members for help if needed. This also shows youth proactive behaviors when dealing with difficult situations. To our knowledge, ours is the first study focusing on the relationship between caregivers’ sources of anxiety and how their children dealt with difficult situations during the COVID-19 pandemic.

Overall, caregivers were very perceptive of their children’s emotional experiences in the previous 2 months with some exceptions: their children felt lonelier, more worried about schoolwork and grades than their caregivers realized. The pandemic social restrictions, as other studies have shown, had a substantial impact on youth emotional states and caregivers were attuned to their children’s emotional changes. This finding is important for researchers and practitioners, as many surveys during the COVID-19 pandemic were directed at caregivers or at youth only. Examining the differences between caregivers’ perceptions of their children’s emotional states and youth’s actual emotional states could be used to further assess, and predict, children’s psychological health.

Almost 1 year after the pandemic, the impact of social restrictions was still raw, even though the U.S. had eased these restrictions and many children were attending school in person, to some degree. As our results show, at least one fourth of children between the ages of 9 and 18 years were experiencing worries and negative emotions often or always. These are alarming numbers that call for urgent interventions and further screening to see if, as school and family routines return to pre-pandemic times, the negative emotional state of our youth decreases. In addition, school closures during the COVID-19 pandemic have implications related to schooling after the pandemic. For example, Commodari and La Rosa (2021) discuss the association of distance learning during the pandemic with students’ distress related to homework, difficulty organizing their study time, focusing on studying, and worries about their future career options. These areas of distress and school-related difficulties will need to be addressed by schools as students return to in-person school. They might also generate new sources of stress as students may feel ill prepared to assume the pre-pandemic learning expectations. This need for support in school is emphasized in the study conducted by Dudovitz et al. (2022) with results suggesting deficits in child wellbeing being related to peer problems, prosocial skills, and the need for learning support and mental wellness. School closures during the COVID-19 pandemic also have implications relating to dietary and sleep habits of children and adolescents, disrupting their routine, which can make it difficult in their schooling after the pandemic (Segre et al., 2021).

Based on the results obtained, we recommend follow up studies to determine whether the negative emotional state of our youth has changed since the return to in-person schooling. In addition, future studies should address areas of distress and school-related difficulties in post-pandemic schooling by potentially implementing interventions to decrease these areas for students. Interventions could consist of a required work-based learning class or an allotted time each school day as a study period. A work-based learning class would allow students to explore career goals, abilities, and interests, which could help students who are worrying about their future career options. An allotted study period would allow students to get a jumpstart on homework, extra help from teachers or other students, and be a time to relax during the school day.

Our study had some limitations. First, it used a convenience sampling approach as the pandemic was still ongoing. Although we advertised through local venues, our sample was recruited mostly using a snowball sampling technique, which led to a sample that was mainly educated, suburban, and middle class. Second, in trying to pair caregivers with their children, we ended up having a small sample of these dyads, making it more difficult to generalize the results to the population. As mentioned in our results, some of our results were underpower after conducting Bonferroni’s adjustments. Lastly, due to the short period available to capture the reality of caregivers and their children, we developed our survey *ad hoc*, using questions from studies in Europe and China, not standardized scales that have higher validity and reliability. Regardless, our study provides further evidence on the effects of the COVID-19 pandemic on youth’s mental health and psychological wellbeing and caregivers’ sources of anxiety and how these two relate. Our results align with results from other studies (e.g., Segre et al., 2021) regarding the psychological effects of the COVID-19 in children and their caregivers and highlight the short-and-long-term impact of this life disruptions to learning and wellbeing.

## Data availability statement

The raw data supporting the conclusions of this article will be made available by the authors, without undue reservation.

## Ethics statement

The studies involving human participants were reviewed and approved by the Institutional Review Board, Ithaca College. Written informed consent to participate in this study was provided by the participants’ legal guardian/next of kin.

## Author contributions

JP-S led the design of the questionnaire, organized the survey, conducted all statistical analyses, and completed the manuscript. AH, CM, and KB carried out the literature searches and manuscript preparation and wrote the first draft of the literature review, methods, and discussion. JP-S, AH, CM, and KB carried out manuscript editing. All authors approved the final version of the manuscript.

## Funding

This study was funded by Ithaca College.

## Conflict of interest

The authors declare that the research was conducted in the absence of any commercial or financial relationships that could be construed as a potential conflict of interest.

## Publisher’s note

All claims expressed in this article are solely those of the authors and do not necessarily represent those of their affiliated organizations, or those of the publisher, the editors and the reviewers. Any product that may be evaluated in this article, or claim that may be made by its manufacturer, is not guaranteed or endorsed by the publisher.

## Supplementary material

The Supplementary material for this article can be found online at: https://www.frontiersin.org/articles/10.3389/fpsyg.2023.1115322/full#supplementary-material
