# Supplementary material for: Caregivers’ anxiety and perception of their children’s wellbeing: a year into the COVID-19 pandemic
Source: Front Psychol. 2023 May 18;14:1115322. doi: 10.3389/fpsyg.2023.1115322 (PMC10234571; doi:10.3389/fpsyg.2023.1115322)
Supplement: Supplementary file 1 [file Table_1.DOCX]

Additional material

*Factor loadings and communalities based on a principal components analysis with oblimin rotation for 12 items from Caregivers Anxiety Scale (*N *=92)*

| Anxious and nervous about the COVID-19 pandemic as it relates to | Children’s development and wellbeing | Finances and personal wellbeing | Children’s use of time | Commonality |
| --- | --- | --- | --- | --- |
| My child(ren)’s mental health. | .88 |  |  | .82 |
| My children’s emotional development. | .87 |  |  | .76 |
| My children’s social development. | .85 |  |  | .74 |
| My children’s school performance. | .65 |  |  | .52 |
| My personal finance. |  | .78 |  | .57 |
| My physical health. |  | .75 |  | .64 |
| My mental health. |  | .71 |  | .58 |
| My personal relationships. |  | .70 |  | .67 |
| Other family members’ health. |  | .64 |  | .54 |
| My children being bored. | .32 |  | .71 | .63 |
| My children spending too much time using screen technology. |  |  | .625 | .40 |
| My children being too busy with school. |  |  | .61 | .45 |
